# Evaluation of an information booklet for adolescents on depression: evidence from a randomized controlled study

**DOI:** 10.1186/s13034-023-00614-x

**Published:** 2023-05-27

**Authors:** Regine Primbs, Lisa Feldmann, Lucia Iglhaut, Antje-Kathrin Allgaier, Gerd Schulte-Körne, Ellen Greimel

**Affiliations:** 1 Department of Child and Adolescent Psychiatry, Psychosomatics and Psychotherapy, Hospital of the Ludwig- Maximilians-University (LMU) Munich, Nussbaumstrasse 5, D-80336 Munich, Germany; 2 Institute of Psychology, Universität der Bundeswehr München, Neubiberg, Germany

**Keywords:** Major depression, Adolescents, Psychoeducation, Knowledge enhancement, Depression literacy, Information booklet

## Abstract

**Background:**

Adolescents with depression often show barriers to seek treatment offers due to various reasons, including limited knowledge about the manifestation of the disorder, its treatment options, or fear of stigmatization. Psychoeducational approaches might reduce these barriers by increasing depression literacy. The aim of the present randomized controlled study was to evaluate whether an innovative and age-appropriate evidence-based information booklet about youth depression increases depression-specific knowledge in adolescents with depression and is also appealing to the target group.

**Methods:**

50 adolescents with a history of depression (current/remitted) aged 12–18 years participated in the study including a pre-, post- and follow-up assessment. Participants were randomly assigned to one of two groups. The experimental group received a target group-specific information booklet about youth depression including seven subdomains. The active control group received an information booklet about asthma in youth that was highly comparable to the depression booklet in terms of format and length. Before and after reading, and at a four-week follow-up, we assessed knowledge about youth depression based on a questionnaire. Furthermore, participants evaluated the acceptability of the information booklets.

**Results:**

Unlike the active control group, the experimental group showed a significant increase in depression-specific knowledge from pre to post and from pre to follow-up across all subdomains. This increase was evident in four subdomains (“symptoms”, “treatment”, “antidepressants”, and “causes”). The overall reception of the information booklet about depression was positive and participants stated that they would recommend the information booklet about depression to their peers.

**Conclusion:**

This is the first randomized controlled study to demonstrate that an information booklet about youth depression effectively imparts depression-specific knowledge to participants with a history of depression and shows high acceptance. Information booklets that are appealing and increase depression-specific knowledge might be a promising low-threshold and cost-effective approach to reduce barriers to treatment and raise awareness.

**Supplementary Information:**

The online version contains supplementary material available at 10.1186/s13034-023-00614-x.

## Background

The risk of developing a depressive disorder markedly increases during adolescence to a 12-month prevalence rate of about 7.5% [1, 2]. Depressive disorders that begin early in life are associated with particularly serious psychosocial and medical consequences [3–5]. A history of depression is an important risk factor for suffering from further episodes [6–8]. Although effective forms of treatment exist, findings suggest that only 12% of adolescents who suffer from depressive symptoms seek professional treatment [9]. There are several barriers against seeking professional treatment services including fear of stigmatization [10–12]. Moreover, affected adolescents and families are often not adequately informed about the symptoms of depression or existing treatment options [13–15]. Improving literacy about the disorder and professional treatment services might help to reduce existing barriers in youth.

Improved depression literacy can be achieved through psychoeducation (e.g., 16; for a review see 15), which can be provided e.g., within campaigns, printed information booklets, or websites. An advantage of psychoeducational material is that it is feasible to implement in different settings, such as school settings, for individuals or within families [17].

A recent systematic review [17] on the efficacy of psychoeducational offers in the context of adolescent depression concluded that these interventions might have beneficial effects on several outcomes, including knowledge about the disorder, attitudes and also depressive symptoms and mental health. As the review identified only few studies which varied considerably with regards to methodological aspects, it was not possible to compute overall effectiveness. Moreover, the authors called for further well-designed studies to draw more comprehensive conclusions [17], including randomized controlled studies. In particular, methodological rigorous studies on the effects of psychoeducation that include youth with clinical diagnoses of depression are very limited and thus represent a research gap.

In line with findings in the field of adolescent depression, studies in adults with depressive symptoms provide evidence that psychoeducation on depression increase depression-specific knowledge [18], reduces depressive symptomatology (for a meta-analysis, see [19]), and has positive effects on the course of depression (for a review, see [20]). Together, these findings illustrate the potential of psychoeducational offers to improve depression literacy and depressive symptomatology in individuals affected by depression. Furthermore, the improvement of mental health literacy is an important determinant for help seeking behavior [21], recognition of depression [22], and destigmatization [23].

One low-threshold psychoeducational approach which can be easily implemented in different settings, is cost-effective and shows high acceptance rates [24], is the use of information booklets. Recently, we developed an innovative, age-appropriate information booklet named “Paul down in the dumps.” which addresses both healthy and adolescents with depression [16, 25]. The booklet aims to increase depression literacy by informing these groups about depression (e.g., symptoms and treatment). In a first pre-post study in healthy adolescents, we found an increase in depression literacy as well as a change in attitude after the reception of the booklet [25]. A second pre-post-follow-up study replicated these results and furthermore found that the increase in knowledge was sustained at a four-week follow-up [16]. Furthermore, this study demonstrated high acceptance rates, which is in line with other studies evaluating information booklets in clinical or healthy youths [24, 26]. Together, the results suggest that an information booklet about depression represents a low-threshold and effective approach to educate healthy adolescents on youth depression. However, as both studies were not randomized controlled studies, it remains an open question whether increased depression literacy can indeed be attributed to the information booklet. Moreover, it remains unknown whether such a booklet is also effective in improving depression literacy among a clinical sample of adolescents with depression.

Building on previous findings [16, 25], the aim of the present study was to investigate whether the aforementioned information booklet effectively increases depression literacy in a clinical sample of adolescents with a history of depression based on a randomized controlled study. We also examined exploratively whether the reception of the information booklet might decrease depressive symptomatology. Finally, we aimed to examine the acceptability of the information booklet. Based on previous results [16, 25], we hypothesized that participants who receive the information booklet about depression would show a higher increase in knowledge about depression than adolescents who receive a control information booklet. We expected this increase in knowledge to occur both immediately after reading the contents and to sustain with stability over time. As an information booklet on depression represents a low-intensive psychoeducational approach, we did not expect effects on depressive symptomatology. Moreover, we hypothesized high acceptance rates of the information booklet [16, 25].

## Methods

### Participants and recruitment

50 adolescents between the age of 12 and 18 years (*M*_age_ = 15.8, *SD*_*age*_ = 1.52) with a history of depression (*n* = 25 current depression, *n* = 25 remitted depression) took part in the study (see flowchart, Additional file 1). The presence of a current or remitted depression based on ICD-10 [27] and potential current or past comorbid disorders were assessed with the standardized interview “Diagnostic Interview for Mental Disorders for Children and Adolescents” (Kinder-DIPS; [28, 29]) administered by a clinician. Participants were classified as remitted if they did not meet the criteria for a depressive episode in the last two months prior to diagnostic assessment based on the “Kinder-DIPS”, as this time span is used to define full remission [30]. Participants completed the Beck Depression Inventory-II (BDI-II; [31]) as self-report to assess the severity of current depressive symptoms. Participants were in- and outpatients of the Department of Child and Adolescent Psychiatry, Psychosomatics and Psychotherapy, Hospital of the Ludwig-Maximilians-University (LMU) Munich. Further inclusion criteria required sufficient German language skills and an intelligence quotient (IQ) ≥ 80. If available, we used IQ information from clinical routine care based on the the Wechsler Intelligence Scale for Children - Fourth Edition (WISC-IV) [32] or the Wechsler Intelligence Scale for Children - Fifth Edition (WISC-V) [33]. If no information from routine care was available, the Culture Fair Intelligence Test-Revised (CFT-20-R) [34] was conducted instead in the context of the present study to obtain information on IQ. Exclusion criteria were acute suicidality, a current or past diagnosis of asthma (as the control information booklet informed about asthma), or a current or past comorbidity with schizophrenic disorder, pervasive developmental disorder, bipolar disorder, or mental and behavioral disorder caused by psychotropic substances. Participants were randomly assigned to one of two groups: The experimental group (EG), who received an information booklet about youth depression; and the active control group (CG), who received an information booklet about asthma in youth. Randomization was based on a predefined list which included a 1:1 randomization stratified by sex and age (< 15 years vs. ≥ 15 years of age). The EG and the CG were comparable in demographic and clinical characteristics (see Table 1). 60% of the participants in both groups had at least one current comorbid psychological disorder on axis I (mainly anxiety disorders).

**Table 1:** Demographic and clinical characteristics of the study sample (N = 50)

	EG (*n* = 25)	CG (*n* = 25)	*p* ^d^
Age (M, SD)	15.86 (1.68)	15.75 (1.37)	0.801
Sex (f/m)	18/7	18/7	0.623
IQ (M, SD)	111.44 (15.38)	114.32 (7.73)	0.408
Depression status (c/r)	16/9	17/8	0.765
BDI-II score (M, SD)^a^	30.44 (15.27)	25.64 (13.19)	0.240
Level of education (lower/higher)^b^	7/18	6/19	0.747
Antidepressant medication (yes/no)^c^	9/16	9/16	0.616

*Note.* BDI-II = Beck Depression Inventory-II. c = current Major Depression Disorder. CG = active control group. EG = experimental group. M = mean. r = remitted Major Depression Disorder. SD = standard deviation

^a^BDI-II score

^b^Level of education: lower = lower secondary education (German “Mittelschule” or “Realschule”). higher = higher academic secondary education (German “Gymnasium”)

^c^Antidepressant medication in the EG: Fluoxetine: *n* = 3, Sertraline: *n* = 3, Citalopram: *n* = 1, Escitalopram: *n* = 1, Bupropion: *n* = 1; Antidepressant medication in the CG: Fluoxetine: *n* = 8, Sertraline: *n* = 1

^d^p-values were obtained from t-test for continuous variables, Fisher’s exact test for binary variable and Chi-squared test for categorial variables

As in the present study, we included questions about the acceptance of the information booklet, we assessed participants’ social desirability with the Social Desirability Scale-17 (SDS-17; [35]). The two groups did not differ in socially desirable response tendencies (*p* = .489). As depression may impact on cognitive functions, like attention [36], we applied the “d2” test (d2; [37]), a well-established paper-pencil instrument to assess concentration and attentional performance. The EG and CG were comparable in their attentional performance (*M*_*EG*_ = 104.32, *SD*_*EG*_ = 9.24, *M*_*KG*_ = 101.52, *SD*_*KG*_ = 9.36, *p* = .292).

The study was approved by the institutional review board and was performed in accordance with the latest version of the Declaration of Helsinki and national legislation. Participants were informed about the aims and procedures of the study and provided written informed assent (participants < 18 years) or written informed consent (18-year-olds). Additionally, for those < 18 years, written informed consent was obtained by at least one parent/legal guardian after they had been informed about all aspects of the study.

### Materials and procedure

#### The information booklet about depression

**Fig. 1:**
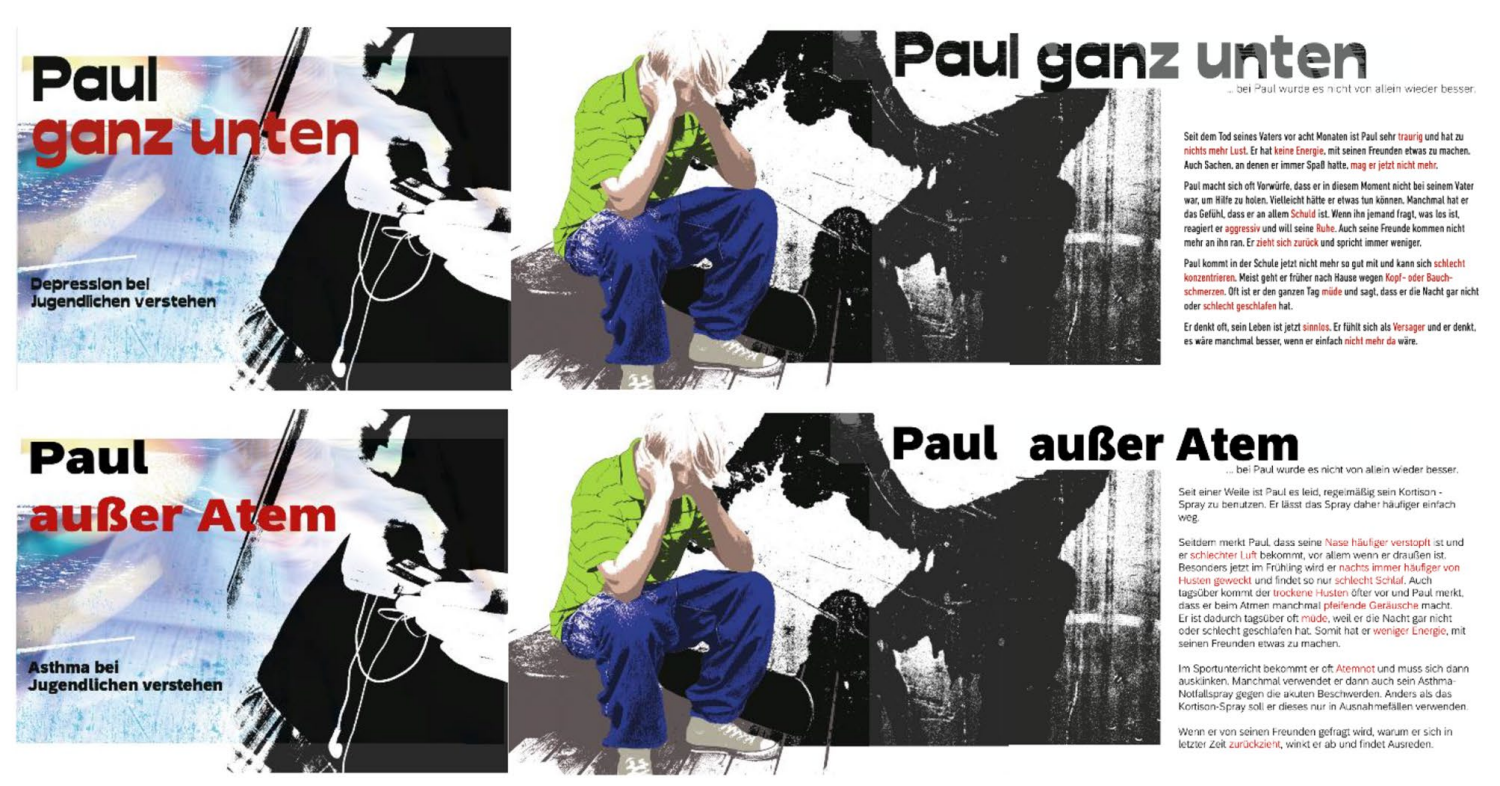
Cover illustrations and the page about information on symptoms of the diseases (written in German). For an English translation of the textual information imbedded in these illustrations, see Additional file 2.

The information booklet about depression (“Paul down in the dumps. Understanding depression in adolescents”; German: “Paul ganz unten. Depression bei Jugendlichen verstehen.”) was developed by our group [16, 25]. It is a twenty-sided hardcopy information booklet, which addresses healthy adolescents as well as adolescents with depression. The storyboard is about Paul who suffers from depressive symptoms. Emilie, his friend, suffered from depression in the past and now supports Paul by offering him advice. The booklet presents evidence-based information on seven depression-related topics in an age-appropriate way (prejudices, symptoms, treatment, antidepressants, causes, suicidality, and helping behavior). The content and layout of the booklet are described in detail elsewhere [16, 25]. Exemplary illustrations of the booklet are shown in Fig. 1.

#### The information booklet about asthma

For this study, we developed a hardcopy control information booklet which informs about asthma as one of the most common chronic somatic conditions in youth [38] (“Paul out of breath. Understanding asthma in adolescents.”; German: “Paul außer Atem. Asthma bei Jugendlichen verstehen.”). Apart from differing contents, both booklets show utmost comparability in relevant aspects: The booklet about asthma has the same format, the same page count, the same illustrations as well as the same multi-color printing as “Paul down in the dumps”. Furthermore, the addressed condition-specific topics are similar (e.g., including symptoms, causes, treatment, helping offers). The narrative in the booklet about asthma is adapted to this specific condition and presents Paul who suffers from asthma-specific symptoms. Emilie’s brother suffered from asthma in the past and she now tries to help Paul by offering him advice (e.g., on healthy lifestyle, avoidance of triggers). As in the booklet about depression, the language of the control information booklet is age-appropriate and easy to understand. For illustrations see Fig. 1.

### Assessment of changes in knowledge

A self-designed knowledge questionnaire based on [25] and [16] was applied to measure both the baseline knowledge (pre-assessment; pre) and changes in knowledge on depression at post-assessment (post) and follow-up assessment (fu). The questionnaire included 48 items, addressing seven different topics, that are included in the information booklet about depression: (1) “prejudices” (4 items, e.g., “Seeking help for depression is a sign of weakness.”); (2) “symptoms” (19 items, e.g., “One is secluding oneself more.”); (3) “treatment” (10 items, e.g., “The psychiatrist or psychotherapist offers help when someone suffers from depression.”); (4) “antidepressants” (3 items, e.g., “When taking antidepressants, you are no longer yourself.”); (5) “causes” (6 items, e.g., “Negative experiences (e.g., death) can contribute to the development of a depression.”); (6) “suicidality” (2 items, e.g., “Suicide is a common cause of death in adolescents”); (7) “helping behavior” (4 items, e.g., “If someone is affected by depression or suicidality, one should listen and offer help.”). The questionnaire used a four-point rating scale (0: not accurate, 1: somewhat accurate, 2: mainly accurate, 3: entirely accurate) with a possible sum score range from 0 to 144 (a higher sum score reflects higher knowledge). In our sample, the internal consistency (Cronbach’s α) for the total knowledge score was 0.45 (pre), 0.78 (post), and 0.70 (fu), respectively. The higher internal consistencies at post and fu compared to pre likely reflects that the questionnaire specifically related to the contents of the booklet, which was presented after the pre-assessment.

### Acceptance of the information booklet

As in [25] and [16], a self-designed evaluation questionnaire was used to assess the layout, content, utility, and overall assessment of both information booklets. The evaluation questionnaire was nearly identical for the EG and CG except that the items referred to depression or asthma, respectively. The questionnaire also used a four-point rating scale. Items are summarized in the Additional file 3.

### Procedure

The pre and post testings took place at the research laboratory of the department. Whenever possible, also the fu testings were conducted at the department. However, due to the corona pandemic, some participants had to be offered the opportunity to fill in the fu questionnaires at home. These participants (*n* = 6 in the EG and *n* = 9 in the CG) did not complete the BDI-II at fu as in our department, the BDI-II is only applied when the presence of professionals is guaranteed. The study design including the assessments is illustrated in the Additional file 4. After diagnostic information had been obtained at a diagnostic appointment (T0; Kinder-Dips, BDI-II), participants were randomly assigned to the EG or CG. At the second appointment, all participants completed the d2 and filled out the self-designed depression-specific knowledge questionnaire (T1a: pre). Participants were then instructed to attentively read the information booklet quietly to themselves (depending on group assignment either the booklet about depression or asthma) in a concentrated manner in the presence of the experimenter. Participants had 15 min to read through the booklet and were allowed to scroll back and forth. If participants indicated that they had read through the booklet before the 15 min were over, they were encouraged to scroll through the booklet once again. After the 15 min reading time, all participants completed the same knowledge questionnaire (T1b: post), which they had completed at pre. In addition, they were asked to fill out the evaluation questionnaire. After four weeks at the third appointment (T2: fu), participants filled out the same knowledge questionnaire as before (pre, post). At T2, depressive symptoms based on the BDI-II were reassessed to allow explorative analyses on the differential effects of the information booklets on depression symptomatology. Participants who received the information booklet about asthma were given the opportunity to receive the information booklet about depression after fu.

### Data analysis

Statistical data analysis was carried out using IBM SPSS Statistics version 26. For all analyses, the significance level was set to *p* = .05 (two-tailed). To obtain knowledge scores for the three time points in both groups, we calculated unweighted index values [39] based on [16]. For a similar approach and details see [16].

To compare baseline knowledge between the EG and CG, independent t-tests were calculated. Changes in total knowledge were analysed using a repeated-measures analysis of variance (ANOVA) for the index value calculated across all seven subdomains (factor time (pre/post/fu) as within-subject factor; factor group (EG/CG) as between-subject factor). To investigate changes in knowledge over time for the subdomains, we calculated a global repeated-measures ANOVA with the within-subject factors subdomain (7 subdomains) and time (pre/post/fu), and group (EG/CG) as the between-subject factor. Thereafter, a follow-up repeated-measures ANOVA with the within-subject factor time (pre/post/fu) and the between-subject factor group (EG/CG) was calculated for each subdomain. In case of significant effects in the repeated-measures ANOVAs, we conducted post-hoc t-tests, thereby applying a Bonferroni-correction for multiple testing. If the sphericity assumption was violated in the ANOVAs, Greenhouse-Geisser’s correction was applied (Mauchly’s test). To exploratively address the question whether baseline knowledge and changes in knowledge in the EG were influenced by depressive symptomatology, sociodemographic or cognitive variables, multiple regressions were conducted (for details and results see the Additional file 5).

To analyse the acceptance of the information booklet about depression in the EG, descriptive statistics (*M, SD*) were calculated. The overall acceptance rate between the EG and CG was compared based on an independent t-test. Spearman’s rank correlations were computed between SDS-17 scores and the answers of the evaluation questionnaire to investigate whether participants’ social desirability was related to participants’ answers. For these analyses, we applied no correction for multiple comparisons, which - in this case - is the more conservative approach with regard to the validity of our results.

In explorative subgroup analyses, we calculated Mann-Whitney U tests to determine if there are any differences in knowledge change over time (pre to post, pre to fu, post to fu) between inpatients (*n* = 8) and outpatients (*n* = 17) in the EG (for details and results see the Additional file 6).

In an explorative analysis, we investigated changes in depressive symptoms based on the BDI-II from pre to fu and ran a 2 × 2 ANOVA with group (EG/CG) as between and time (pre/fu) as within-subjects factor. Due to some missing data for the BDI-II at fu (6 out of 25 in the EG, 9 out of 25 in the CG, for details see “Procedure”), this analysis was restricted to a subsample of *n* = 35 participants.

### Power analysis

To determine the necessary sample size to test our main hypothesis of knowledge gain in the EG from pre to post and from pre to fu, a priori power analysis was computed. As there are no other comparable studies evaluating an information booklet within a randomized controlled study design, we based our analysis on a previous study [26], which examined knowledge gain in youth after reading an information booklet about psychotherapy and mental disorders applying a pre-post-fu design. The authors found large effect sizes for knowledge gain from pre to post, as well as from pre to fu (*d* ≥ 1.13). Based on these results and a conservative assumption of only medium to large effect sizes in adolescents with a history of depression who presumably have prior knowledge about depression, *n* = 24 participants in the EG are needed to detect these effects (assuming an alpha error of 0.05 and a power of 0.80). Thus, the sample of the present study with *n* = 25 participants in the EG was sufficiently large to detect the expected effects.

## Results

### Baseline knowledge about depression

**Fig. 2:**
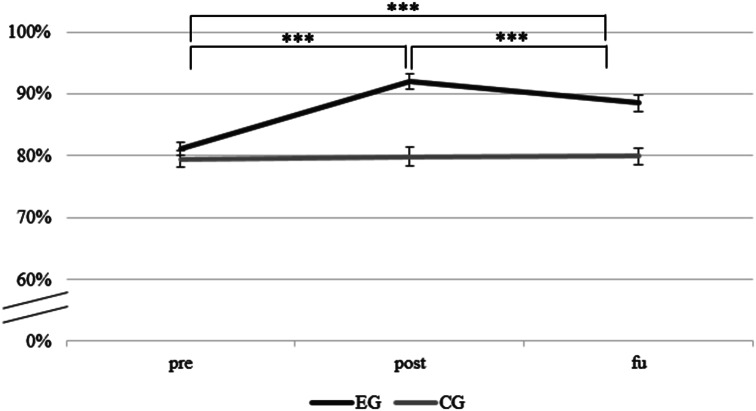
Changes in total knowledge over time (M, SE). ***p < .001. CG = active control group. EG = experimental group. fu = follow-up assessment after 4 weeks. M = mean. post = post-assessment. pre = pre-assessment. Error bars show standard errors (SE). The Y-axis depicts knowledge score. Corrected *p*-values for six tests (0.05/6 = 0.008).

**Fig. 3:**
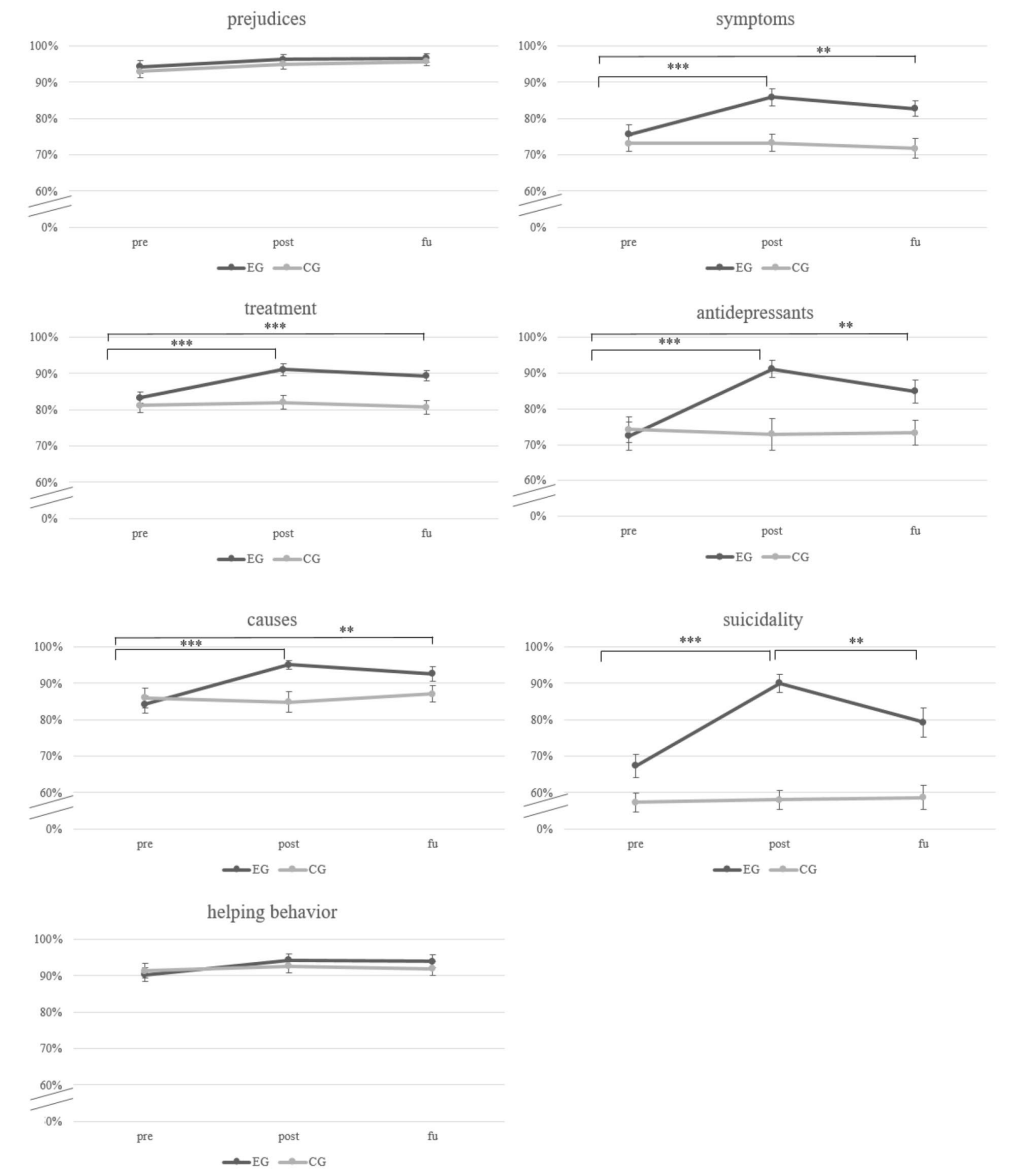
Changes in knowledge over time in both groups in the different subdomains (M, SE). **p < .01, ***p < .001. CG = active control group. EG = experimental group. fu = follow-up assessment after 4 weeks. M = mean. post = post-assessment. pre = pre-assessment. Error bars show standard errors (SE). The Y-axis depicts knowledge score.

Participants’ total baseline knowledge was comparable in the two groups (EG: *M* = 80.56%, *SD* = 9.73; CG: *M* = 79.03%, *SD* = 10.50; *t*(48) = − 0.94, *p* = .351, *d* = 0.27 ) (see Fig. 2). This was also the case for the subdomains (all *p*s ≥ 0.385), except for the subdomain suicidality (*t*(48) = −2.48, *p* < .05, *d* = 0.7), where participants in the EG showed higher baseline scores (see Fig. 3).

### Changes in knowledge over time

The 2 × 3 repeated-measures ANOVA on changes in total knowledge across all subdomains revealed a significant main effect of time, *F* (1.75, 84.07) = 39.05, *p* < .001, partial *η²* = 0.45, a significant main effect of group, *F*(1, 48) = 19.57, *p* < .001, partial *η²* = 0.29, as well as a significant interaction between time and group, *F* (1.75, 84.07) = 33.77, *p* < .001, partial *η²* = 0.41.

Post-hoc dependent samples t-tests (corrected p-values for six tests: 0.05/6 = 0.008) revealed a significant increase in knowledge in the EG from pre to post (*t*(24) = −10.07, *p* < .001, *d* = 2.0) and from pre to fu (*t*(24) = −5.67, *p* < .001, *d* = 1.13). The comparison of index values between post and fu was significant in the direction that the knowledge decreased (*t*(24)= −4.111, *p* < .001, *d* = 0.82). In the CG, none of the post-hoc tests reached significance with all *p*s ≥ 0.501 (see Fig. 2).

The 7 × 3 × 2 global repeated-measures ANOVA on changes in knowledge over time for the seven subdomains showed a significant interaction effect between subdomain, time, and group, *F* (5.3, 253.1) = 3.51, *p* < .01, partial *η²* = 0.07. We found a significant main effect of time, *F* (1.75, 84.07) = 39.05, *p* < .001, partial *η²* = 0.45, a significant main effect of group, *F*(1, 48) = 19.57, *p* < .001, partial *η²* = 0.29, as well as a significant main effect for the subdomains, *F* (4.14, 198.53) = 56.03, *p* < .001, partial *η²* = 0.54.

The follow-up repeated-measures ANOVAs on changes in knowledge over time for the seven subdomains revealed significant interaction effects between time and group for all subdomains, except for the subdomains “prejudices” and “helping behavior” (see Table 2). Significant main effects of time were found for all subdomains, except the subdomains “prejudices” and “helping behavior”; significant main effects of group were found in all subdomains, except for the subdomains “prejudices”, “causes”, and “helping behavior”(see Fig. 3).

**Table 2:** Results on interaction and main effects of the ANOVAs

subdomains	*F*	*df*	*partial η²*	*p*
*Interaction between time and group*				
Prejudices	0.02	1.65	.00	.970
Symptoms	9.08	2	.16	< .001
Treatment	8.36	2	.15	< .001
Antidepressants	10.02	2	.17	< .001
Causes	10.91	2	.19	< .001
Suicidality	9.00	1.77	.16	< .001
Helping behavior	1.25	2	.03	.292
***Factor time***				
Prejudices	3.06	1.65	.06	.062
Symptoms	8.28	2	.15	< .001
Treatment	10.30	2	.18	< .001
Antidepressants	7.53	2	.14	< .001
Causes	9.29	2	.16	< .001
Suicidality	10.19	1.77	.17	< .001
Helping behavior	3.71	2	.07	.028
***Factor group***				
Prejudices	0.57	1	.01	.453
Symptoms	8.31	1	.15	< .01
Treatment	9.19	1	.16	< .01
Antidepressants	4.65	1	.09	.036
Causes	2.66	1	.05	.110
Suicidality	45.57	1	.49	< .001
Helping behavior	0.15	1	.003	.703

*Note.* F-test statistic of the ANOVA, *df* = degrees of freedom, *partial η²* = effect size of the ANOVA

The significant interaction effects between time and group within five subdomains (all subdomains except “prejudices” and “helping behavior”) were followed-up by repeated-measures ANOVAs with the within-factor time (pre/post/fu), separately for the two groups (EG/CG). In the EG, significant effects of time were revealed for all of these five subdomains: “symptoms”, “treatment”, “antidepressants”, “causes”, and “suicidality” (all *p*s < 0.001). In the CG, the follow-up ANOVAs revealed no significant effects of time (with all *p*s ≥ 0.300). Thus, we did not conduct further post-hoc-comparisons for the CG.

The post-hoc dependent t-tests to follow-up the significant effects of time in the EG revealed a significant increase in knowledge from pre to post in the EG in the subdomains “symptoms”, “treatment”, “antidepressants”, “causes”, and “suicidality” (all *p*s < 0.001). The increase for the subdomain suicidality had the largest effect size (*d* = 1.06). Moreover, there was a significant increase in knowledge from pre to fu for the subdomains: “symptoms”, “treatment”, “antidepressants”, and “causes” (all *p*s < 0.01). The increase in knowledge from pre to fu for the subdomain “suicidality” did not withstand correction for multiple testing (*p* = .031; corrected p-values for five tests: 0.05/5 = 0.01). From post to fu, no significant changes in knowledge occurred (all *p*s ≥ 0.078) with one exception: there was a significant decrease in knowledge for the subdomain “suicidality” (*p* < .01) (see Fig. 3).

### Acceptance of the information booklets

Results on the overall assessment of the information booklet about depression showed very good to good ratings (*M* = 1.72 on a rating scale from “1 - very good” to “6 - insufficient”). The overall assessment of the information booklet about asthma revealed lower ratings (*M* = 2.28 on a rating scale from “1 - very good” to “6 - insufficient”), *t*(48) = 2.59, *p* < .05, *d* = 0.72. The Additional file 3 summarizes the results for the items of the evaluation questionnaire of the information booklet about depression. For the EG, correlation analyses showed that social desirability was unrelated to participants’ response behavior concerning the items of the evaluation questionnaire with all *p*s ≥ 0.079.

### Changes in depressive symptoms

The exploratory analysis on changes in depressive symptoms based on the BDI-II revealed neither a significant main effect of time (*F*(1, 33) = 2.03, *p* = .163, partial *η²* = 0.058) and group (*F*(1, 33) = 0.90, *p* = .349, partial *η²* = 0.027) nor a significant interaction (*F*(1, 33) = 0.13, *p* = .719, partial *η²* = 0.004). Descriptive parameters suggested that both groups showed a slight decline in depressive symptoms over time (EG: *M*_*pre*_ = 28.95, *SD*_*pre*_ = 15.03; *M*_*fu*_ = 27.16, *SD*_*fu*_ = 15.07; CG: *M*_*pre*_ = 24.00, *SD*_*pre*_ = 14.51; *M*_*fu*_ = 22.94, *SD*_*fu*_ = 13.24).

## Discussion

This randomized controlled study aimed to investigate whether an information booklet on youth depression was effective in enhancing depression literacy in adolescents with a history of depression. A second main aim was to evaluate the acceptance of the information booklet. In line with our expectations, the information booklet about depression effectively enhanced depression literacy in adolescents with a history of depression both short- and long-term. We found an increase in knowledge from pre to post for the subdomains symptoms, treatment, antidepressants, causes, and suicidality, which remained stable after a period of one month except for the subdomain suicidality. The overall evaluation of the information booklet about depression was positive.

This is the first randomized controlled study that shows an increased depression literacy after the reception of an information booklet about depression in adolescents with a history of depression. Our findings replicate and expand the results from [25] and [16]. In contrast to [16], we found no knowledge increase for the subdomains prejudices (referred to as “depression as a disorder” by 16) and helping behavior. It is conceivable that adolescents with a history of depression have more prior knowledge with regard to these depression-specific content domains compared to healthy youth. Indeed, for these two domains, baseline knowledge was highest, thus absent knowledge gain likely can be attributed to ceiling effects.

Of importance, adolescents in the present study had the lowest baseline knowledge in the subdomain “suicidality”, suggesting comparably little knowledge in this field. In this context, it should be noted that participants in the EG had the highest knowledge gain from pre to post in this subdomain, but that knowledge dropped for this domain from post to fu. Our results should be interpreted with some caution since only two items in our knowledge questionnaire addressed the subdomain suicidality. This having said, suicidality is a crucial topic in the context of depression and it seems particularly important to educate adolescents with a history of depression on suicidality and to consider the relevance of repetition, e.g., in the context of booster approaches, to achieve stable knowledge gain. In this regard, professional treatment offers might be paralleled by low-threshold offers, including evidence-based websites that can be easily accessed. Indeed, there are studies in adults which provide evidence that websites about suicide prevention lead to increased knowledge about preventive aspects [40].

Our explorative findings of larger knowledge enhancement in younger participants as well as in participants with lower IQ suggest that these individuals profit more from the depression-specific information provided. This information can be useful to inform professionals on implementation decisions regarding the information booklet. Moreover, the larger knowledge gain in participants with lower IQ contradicts the notion that text-based psychoeducation is particularly beneficial for those with higher cognitive skills. This being said, it should be noted that the IQ of participants in the EG was in the upper average range. Future studies should replicate our results in a more representative sample regarding cognitive skills. Interestingly, explorative subgroup analyses on differences in knowledge change between inpatients and outpatients suggests that knowledge change was more sustained in inpatients compared to outpatients. This result might perhaps be due to less distraction in an inpatient compared to an outpatient setting and it would be worthwhile to follow up this explorative finding in future studies.

Our findings of the positive evaluation of the information booklet about depression corroborate the results of our previous studies in healthy adolescents [16, 25]. Although our study population had prior experience with treatment of depression in clinical care, the content was highly accepted and they did not feel unnecessarily or overly instructed. This high level of acceptance is an important prerequisite to ensure that the content provided is consumed and that knowledge gain might occur. Our information booklet thus can be seen as a good benchmark and an age-appropriate and appealing psychoeducative approach. It should be noted that the overall acceptance of the control booklet was slightly lower than the depression booklet. This might well be explained by the fact that the content of the control booklet was less relevant to the adolescents with a history of depression.

Albeit not the focus of the present study, our explorative findings suggested that the information booklet on depression had no specific effect on depressive symptoms. While previous research in non-clinical samples has concluded that psychoeducational interventions can have beneficial effects on depressive symptoms [19], the reception of an information booklet is very likely not intense enough to achieve improvements in depressive symptomatology. This might particularly be the case for a clinical sample like the one included in the current study.

### Limitations and Strengths

Some limitations of the study should be noted. Our sample size mainly consisted of females which limits the generalizability of our findings to boys. This having said, the sex ratio in our sample mirrors the female preponderance of depression in adolescence [41, 42] and the regression analyses showed that sex did not influence baseline knowledge or knowledge gain. However, in future studies, it would be important to include more male participants to allow for sex-specific analyses. A further limitation is that we did not evaluate if adolescents applied the newly acquired knowledge and thus profited from it in everyday life. Thus, to be able to draw more stringent conclusions about the transfer of the acquired knowledge into real life, future studies should address this aspect, e.g., by including questions about behavioral intentions regarding help seeking or to assess help seeking behavior after a longer follow-up period. Another option would be to apply ecological momentary assessment methods, which would enable to assess whether participants change their habits and, e.g., talk to friends if they feel depressed. Moreover, our participants were all patients who have been treated in routine care and had prior experiences with treatment of depression. It might well be the case that this sample characteristic might have influenced our study results and that effects regarding knowledge change are even larger in treatment-naïve samples. Thus, in future investigations, it would be of particular interest to include participants with no prior treatment experiences.

Despite these limitations, our study has many strengths. This includes the recruitment of a clinically very well-characterized and sufficiently large patient sample with a history of depression. It should further be emphasised that the control information booklet was highly comparable to the booklet about depression in various important aspects. In this context, unlike in many other studies, knowledge gain was not evident within our active control group and the randomized controlled study design allowed us to draw robust conclusions regarding the efficacy of the information booklet about depression to increase depression literacy.

## Conclusions

The present study provides evidence that the information booklet about depression effectively improves depression literacy in participants with current or remitted depression. An important next step in future studies would be to compare the effectiveness of information booklets with other psychoeducational methods based on RCTs to allow even stronger conclusions regarding the approach taken in the present study (see 43 for a comparative study in youth with alcohol-related problems). Our results also provide important implications for the design of future psychoeducational materials in the context of youth depression. Given the importance of early treatment of depressive disorders in youth [44, 45], in future studies, it would be worthwhile to explore whether information booklets like the one studied here might increase treatment adherence in patients with depression. Another important next step would be to evaluate whether presenting the depression-specific knowledge of our information booklet in a digital format is even more effective given that children and adolescents grow up as digital natives and online formats are gaining increasing importance [46–48]. Moreover, future studies might benefit from including parents in studies on the effectiveness of psychoeducational approaches in youth as research shows that parental mental health literacy improves treatment outcome and help-seeking behaviour of their children [49, 50]. As shown by studies in adolescents, psychoeducational low-threshold information improves the motivation to seek help [26, 51, 52]. In this context, to explore to what extent improved knowledge about depression-specific content also leads to changes in behavior, future studies would benefit from extending their focus to the application of the acquired knowledge in everyday life. Connected to this aspect, it would be important in future studies to include an at-risk sample with no prior contact to professional treatment to obtain insight if barriers to treatment indeed can be reduced.

## Electronic supplementary material

Below is the link to the electronic supplementary material.

Supplementary Material 1

Supplementary Material 2

Supplementary Material 3

Supplementary Material 4

Supplementary Material 5

Supplementary Material 6

## Data Availability

Our data includes sensitive patient information, such as information on comorbidities. Since participants could possibly be identified by making our raw data publicly available, ethical principles of protecting patient confidentiality would be breached. Thus, raw data cannot be made publicly available. We can make additional materials and aggregated data available upon request.

## List of abbreviations

ANOVA: Analysis of variance
BDI-II: Beck Depression Inventory-II
c: Current Major Depression Disorder
CFT-20-R: Culture Fair Intelligence Test-Revised
CG: Active control group
d2: “d2” test
df: Degrees of freedom
EG: Experimental group
fu: Follow-up-assessment
IQ: Intelligence quotient
Kinder-DIPS: Diagnostic Interview for Mental Disorders for Children and Adolescents
LMU: Ludwig-Maximilians-University
M: Mean
pre: Pre-assessment
post: Post-assessment
r: Remitted Major Depression Disorder
SD: Standard deviation
SDS-17: The Social Desirability Scale-17
SE: Standard error
WISC-IV: Wechsler Intelligence Scale for Children - Fourth Edition
WISC-V: Wechsler Intelligence Scale for Children - Fifth Edition
