# Supplementary material for: Evaluation of an information booklet for adolescents on depression: evidence from a randomized controlled study
Source: Child Adolesc Psychiatry Ment Health. 2023 May 27;17:65. doi: 10.1186/s13034-023-00614-x (PMC10225101; doi:10.1186/s13034-023-00614-x)
Supplement: Supplementary file 1 — Supplementary Material 1 [file 13034_2023_614_MOESM1_ESM.docx]

**Additional file 1**

Flowchart illustrating sample selection for the analysis presented in the manuscript text.


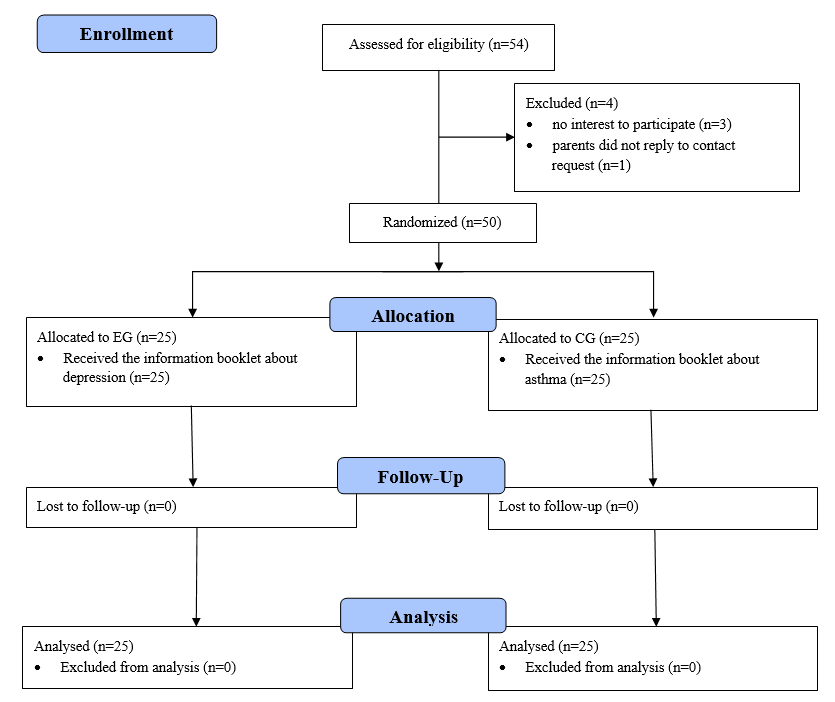


*Abbreviations:*  EG = experimental group. CG = active control group.
