# Supplementary material for: Evaluation of an information booklet for adolescents on depression: evidence from a randomized controlled study
Source: Child Adolesc Psychiatry Ment Health. 2023 May 27;17:65. doi: 10.1186/s13034-023-00614-x (PMC10225101; doi:10.1186/s13034-023-00614-x)
Supplement: Supplementary file 2 — Supplementary Material 2 [file 13034_2023_614_MOESM2_ESM.docx]

**Additional file 2**

**English translation of the textual information imbedded in Fig. 1**

Information booklet on depression

Paul down in the dumps - Paul did not get better on his own. Since his father’s death eight months ago, Paul has been in a depressed mood and has been showing diminished interest and pleasure. He has not been having any energy to do something with his friends. He has also lost interest in things he had usually enjoyed before.

Paul often feels guilty for not having been with his father in that moment to get help. Perhaps he could have done something. Sometimes, he has the feeling that he is responsible for everything that went wrong. When someone asks him what is going on, he reacts aggressively and wants to be left alone. Even his friends cannot get close to him anymore. He withdraws and talks less and less.

Paul struggles to keep up at school and to concentrate properly. He usually leaves school earlier due to headache or stomachache. He is frequently tired the whole day and says that he didn´t sleep at night or slept poorly.

He often thinks that his life is senseless now. He feels like a looser and thinks that sometimes, it would be better if he wasn´t there anymore.

Information booklet on asthma

Paul out of breath – Paul did not get better on his own. For a while, Paul has been tired of using his cortisone spray regularly. Thus, he has started to often just skip to use the spray.

Since then, Paul has noticed that his nose is blocked more often and that he is not able to breathe properly, especially when he is outside. Particularly now in spring, he is waking up more and more often at night due to coughing and thus has trouble sleeping. The dry cough also frequently occurs during the day and Paul has noticed that sometimes, he makes whistling noises when he breathes. As a result, he is often tired during the day because he has not slept at all or has slept poorly during the night. Thus, he has less energy to do something with his friends.

He often gets out of breath in physical education classes and then has to take a break. Sometimes, he then uses his emergency asthma spray against the acute symptoms. Unlike the cortisone spray, he should only use this one in exceptional cases.

When his friends ask Paul why he is withdrawing, he waves aside and finds excuses.
