# Supplementary material for: Evaluation of an information booklet for adolescents on depression: evidence from a randomized controlled study
Source: Child Adolesc Psychiatry Ment Health. 2023 May 27;17:65. doi: 10.1186/s13034-023-00614-x (PMC10225101; doi:10.1186/s13034-023-00614-x)
Supplement: Supplementary file 3 — Supplementary Material 3 [file 13034_2023_614_MOESM3_ESM.docx]

**Additional file 3**

Results for items of the evaluation questionnaire of the information booklet about depression (in %).

| Items | entirely accurate | | mainly accurate | | somewhat accurate | | not accurate | |
| --- | --- | --- | --- | --- | --- | --- | --- | --- |
| I like the pictures in the information booklet. | | 36 | **44** | 12 | | 8 | |  |
| The information booklet is written in a complicated way. | | 0 | 0 | 0 | | **100** | |  |
| Through Paul's story, you can imagine what depression looks like. | | **48** | 36 | 16 | | 0 | |  |
| The amount of information is exactly appropriate. | | **64** | 24 | 8 | | 4 | |  |
| I find the texts in the information booklet interesting. | | **60** | 20 | 16 | | 4 | |  |
| I now know better what I can do about the depression. | | **48** | 20 | 12 | | 20 | |  |
| The information booklet eliminates prejudices about depression. | | **72** | 24 | 4 | | 0 | |  |
| I would recommend the information booklet to other young people who are suffering from depression. | | **52** | 28 | 16 | | 4 | |  |

*Note.* Most frequent answers are shown in bold letters; Using a four-point rating scale (0: not accurate, 1: somewhat accurate, 2: mainly accurate, 3: entirely accurate); *%*; *n* = 25.
