# Supplementary material for: Evaluation of an information booklet for adolescents on depression: evidence from a randomized controlled study
Source: Child Adolesc Psychiatry Ment Health. 2023 May 27;17:65. doi: 10.1186/s13034-023-00614-x (PMC10225101; doi:10.1186/s13034-023-00614-x)
Supplement: Supplementary file 4 — Supplementary Material 4 [file 13034_2023_614_MOESM4_ESM.docx]

**Additional file 4**


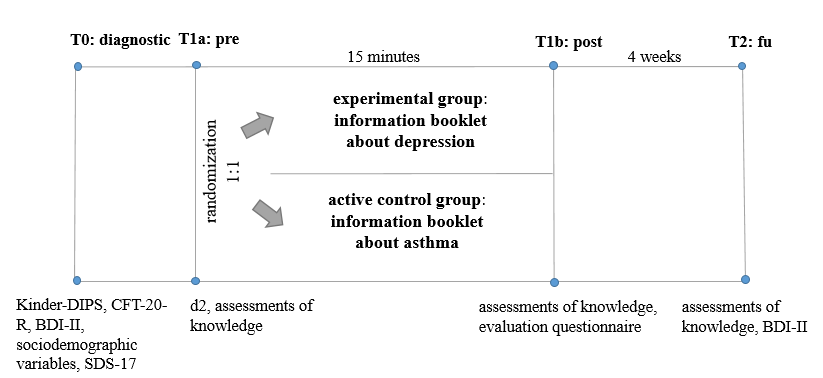
Study procedure

*Note.* BDI-II = Beck Depression Inventory-II. CFT-20-R = Culture Fair Intelligence Test-Revised. d2 = d2 test. fu = follow-up assessment after 4 weeks. Kinder-DIPS = Diagnostic Interview for Mental Disorders for Children and Adolescents. SDS-17 = The Social Desirability Scale-17. post = post-assessment. pre = pre-assessment.
