# Supplementary material for: Evaluation of an information booklet for adolescents on depression: evidence from a randomized controlled study
Source: Child Adolesc Psychiatry Ment Health. 2023 May 27;17:65. doi: 10.1186/s13034-023-00614-x (PMC10225101; doi:10.1186/s13034-023-00614-x)
Supplement: Supplementary file 5 — Supplementary Material 5 [file 13034_2023_614_MOESM5_ESM.docx]

**Additional file 5**

**Prediction of baseline knowledge and knowledge change from pre to post in the experimental group**

To address the question whether baseline knowledge in the EG was influenced by depressive symptomatology, sociodemographic or cognitive variables, a multiple regression (enter method) was conducted with BDI-II score, age, level of education, attentional performance, and IQ as predictors and total baseline knowledge as the criterion. Likewise, to analyse the influence of these predictors on changes in total knowledge from pre to post, a multiple regression (enter method) was computed with the above-mentioned predictors and the difference score of total knowledge (post minus pre) as the criterion.

The results of the regression analysis with BDI-II score, age, level of education, attentional performance, and IQ as predictors and baseline knowledge as the dependent variable showed that the variables had no significant influence on the baseline knowledge and did not account for a significant proportion of variance (*F*_5,19_ = 2.34; *p* = .081; *R^2^* = .38).

The second regression analysis with BDI-II score, age, level of education, attentional performance, and IQ as predictors and knowledge enhancement pre/post as the dependent variable showed that the model accounted for a significant proportion of variance regarding knowledge change from pre to post (*F*_5,19_ = 4.75; p < .01; R^2^ = .56). While age and IQ were significant predictors of knowledge enhancement, level of education, BDI-II score, and attentional performance were not (see **Additional Table** below). More specifically, younger participants exhibited larger knowledge enhancement than older participants. Furthermore, participants with lower IQ had a higher knowledge gain than participant with higher IQ.

**Additional Table.** Results of the regression analyses predicting knowledge enhancement from pre to post

|  | | *B* | *SE for B* | *95 % CI for B* | *β* | *t* | *p* |
| --- | --- | --- | --- | --- | --- | --- | --- |
| BDI-II score | -0.1 | | 0.1 | [-0.4, 0.1] | -.28 | -1.4 | .178 |
| Age | -3.1 | | 0.8 | [-4.8, -1.3] | -.66 | -3.7 | .002 |
| level of education (lower vs. higher) | -0.4 | | 3.5 | [-7.7, 7.0] | -.02 | -0.1 | .920 |
| IQ | -0.3 | | 0.1 | [-0.5, -0.1] | -.61 | -3.2 | .005 |
| attentional performance | 0.3 | | 0.2 | [-0.1, 0.6] | .31 | 1.6 | .128 |
