# Supplementary material for: Evaluation of an information booklet for adolescents on depression: evidence from a randomized controlled study
Source: Child Adolesc Psychiatry Ment Health. 2023 May 27;17:65. doi: 10.1186/s13034-023-00614-x (PMC10225101; doi:10.1186/s13034-023-00614-x)
Supplement: Supplementary file 6 — Supplementary Material 6 [file 13034_2023_614_MOESM6_ESM.docx]

**Additional file 6**

**Subgroup analyses for differences in knowledge change over time between inpatients and outpatients in the experimental group**

To address the question if there are any differences in knowledge over time between the inpatients and the outpatients in the EG, we used the Mann-Whitney U test.

Differences in knowledge change from pre to post between the inpatients (*Mdn* = 18.08) and the outpatients (*Mdn* = 12.67), *U* = 50.00, *z* = -1.05, *p* = .294, *r* = -0.21, were non-significant. Moreover, the difference in knowledge change from pre to fu between the inpatients (*Mdn* = 14.52) and the outpatients (*Mdn* = 8.61), *U* = 37.00, *z* = -1.81, *p* = .071, *r* = -0.36, also failed to be significant. However, we found a significant difference in knowledge change between the inpatients (*Mdn* = -0.87) and the outpatients (*Mdn* = -5.80) from post to fu, *U* = 29.00, *z* = -2.27, *p* = .023, *r* = -0.45, with inpatients showed a smaller decrease in knowledge.
